# Characterization of Intra-Type Variants of Oncogenic Human Papillomaviruses by Next-Generation Deep Sequencing of the E6/E7 Region

**DOI:** 10.3390/v8030079

**Published:** 2016-03-14

**Authors:** Enrico Lavezzo, Giulia Masi, Stefano Toppo, Elisa Franchin, Valentina Gazzola, Alessandro Sinigaglia, Serena Masiero, Marta Trevisan, Silvana Pagni, Giorgio Palù, Luisa Barzon

**Affiliations:** 1 Department of Molecular Medicine, University of Padova, via A. Gabelli 63, 35121 Padova, Italy; enrico.lavezzo@unipd.it (E.L.); giulia.masi@unipd.it (G.M.); stefano.toppo@unipd.it (S.T.); elisa.franchin@unipd.it (E.F.); gazzola.valentina@virgilio.it (V.G.); seremasi@libero.it (S.M.); marta.trevisan@unipd.it (M.T.); silvana.pagni@unipd.it (S.P.); luisa.barzon@unipd.it (L.B.); 2 Veneto Institute of Oncology IOV IRCCS, via Gattamelata 64, 35128 Padova, Italy; alessandrosinigaglia@yahoo.it

**Keywords:** human papillomavirus, genotyping, next-generation sequencing, E6/E7, subtype, variant, cervical cancer, high-risk HPV, deep sequencing, pyrosequencing

## Abstract

Different human papillomavirus (HPV) types are characterized by differences in tissue tropism and ability to promote cell proliferation and transformation. In addition, clinical and experimental studies have shown that some genetic variants/lineages of high-risk HPV (HR-HPV) types are characterized by increased oncogenic activity and probability to induce cancer. In this study, we designed and validated a new method based on multiplex PCR-deep sequencing of the E6/E7 region of HR-HPV types to characterize HPV intra-type variants in clinical specimens. Validation experiments demonstrated that this method allowed reliable identification of the different lineages of oncogenic HPV types. Advantages of this method over other published methods were represented by its ability to detect variants of all HR-HPV types in a single reaction, to detect variants of HR-HPV types in clinical specimens with multiple infections, and, being based on sequencing of the full E6/E7 region, to detect amino acid changes in these oncogenes potentially associated with increased transforming activity.

## 1. Introduction

Papillomaviruses are small circular double-stranded DNA viruses that preferentially infect the basal layer of skin and mucosal epithelia. They are highly species-specific and have been identified in a broad variety of animal species, including reptiles, birds, and mammals [1]. To date, nearly 200 different human papillomavirus (HPV) types have been identified [2,3], including twelve classified as oncogenic or “high-risk” HPV types (HR-HPV) [4]. Different papillomavirus types are defined by the presence of 10% or more nucleotide differences between two phylogenetically close genomes that share a common ancestor, while papillomavirus genomes that differ between 10% and 2% are defined as intra-type variants (also defined as “subtypes” or “lineages”) [5,6]. The high genetic diversity of papillomaviruses is the result of a long evolutionary history with the sequential accumulation of genetic changes over millions of years. Actually, papillomaviruses are characterized by a very low mutation rate because they exploit host replication machinery and proofreading activity to replicate their own genome.

Differences in tissue tropism and ability to promote cell proliferation and transformation have been demonstrated among papillomavirus types. As for HPV types, 12 (*i.e.*, HPV16, HPV18, HPV31, HPV33, HPV35, HPV39, HP45, HPV51, HPV52, HPV56, HPV58, HPV59) have been classified as oncogenic types because of the experimental and/or clinical evidence of their etiological role in the development of cervical cancer and of a subset of anogenital and head and neck squamous cell carcinomas [4]. HPV68, defined as a possible oncogenic HPV type because of the mechanistic evidence of a role in cervical tumorigenesis [4], is generally included among HR-HPV types in diagnostic assays [7].

Differences in oncogenic activity have been demonstrated among HR-HPV types (e.g., HPV16 is the most potent HPV type, able to cause cancer at different anatomic sites, at variance with the other oncogenic HPV types that have been associated with cervical cancer but rarely with other types of cancer). In addition, clinical and experimental studies have shown that the presence of nucleotide changes in some HPV intra-type variants may be associated with increased oncogenic activity and probability to induce cancer [6,8,9,10,11]. For example, non-European lineages of HPV16 have been associated with an increased risk of persistent viral infection and development of cervical precancer and cancer amongst European populations [11,12,13,14]; A1 sublineage of HPV33, B2 sublineage of HPV45, and A1 and A3 sublineages of HPV58 have been found to be overrepresented in cervical cancer [9,10,11].

While further studies are required to confirm the significance of HPV intra-type genetic variants in conferring an increased risk of cancer development, especially for non-HPV16/18 oncogenic types [6], the available data from the literature indicate that identification of HPV types and variants in HPV-positive subjects may be useful for risk stratification and management strategies [9]. So far, characterization of HPV genetic variants in clinical samples has been performed by polymerase chain reaction (PCR) followed by conventional Sanger sequencing of the complete viral genome or of a specific region of the genome (e.g., the L1 ORF, the E6/E7 ORFs, or the long control region) [9,10,13,15,16,17]. While being highly accurate, this technique does not allow testing samples with infection by multiple HPV types, which account for approximately 30% of genital HPV infections [18,19]. In addition, reported methods based on PCR-Sanger sequencing generally require prior knowledge of the infecting HPV type since they use HPV type-specific primers for PCR [9,10]. These technical hurdles can be overcome by using massively parallel high-throughput sequencing (or NGS, next-generation sequencing) technologies, which allow sequencing of multiple HPV types and variants in clinical specimens and the identification of minority genetic variants in individual samples [20,21,22,23,24,25,26,27,28,29]. Reported NGS-based methods for HPV detection and typing consisted either in deep sequencing of amplicons obtained from the amplification of highly conserved regions in HPV genome or in the unbiased metagenomics sequencing of whole DNA or RNA from biological samples. Methods based on the first approach have been developed and validated as diagnostic tests for HPV genotyping [21,22,23,24,25,28,29], while the metagenomics approach has been applied to the study of the molecular mechanisms of virus-related tumors and has led to the discovery on new tumor viruses, including new HPV types [26,27,30,31,32,33].

In this study, we designed and validated a new method for the characterization of intra-type variants of HR-HPVs based on deep sequencing of the whole E6/E7 region. Potential applications of this method include epidemiological studies on the distribution of HPV lineages, investigation of HPV-related cancers to identify HPV variants associated with increased oncogenic potential, and risk-stratification in patients with HR-HPV infection.

## 2. Materials and Methods

### 2.1. Primer Design

A bioinformatics pipeline was implemented with the aim of generating a set of PCR primers able to amplify the whole E6/E7 region of all HPV types classified as oncogenic groups 1 and 2A by the International Agency for Research on Cancer (IARC) [4] in order to identify HR-HPV intra-type variants (or subtypes). The E6/E7 region was chosen as target sequence because it allows a better discrimination of HPV subtypes than other regions of HPV genome [6].

The setup of the pipeline developed for this purpose allowed to two distinct tasks to be accomplished: (1) the possibility to detect and amplify all HR-HPV types in the same reaction and (2) the robustness of the design to the effect of HPV genome variability, at least for all known variants that are deposited in the Nucleotide Database.

To this aim, all nucleotide sequences belonging to HR-HPV types (IARC group 1 and 2A) were downloaded from GenBank (Table 1). For each HPV type, a multiple sequence alignment was generated with USEARCH [34] and visually inspected with Jalview [35], in order to correct alignment artifacts and discard sequences not covering the E6/E7 region (totally or partially). Then, multiple sequence alignments were scanned column by column and all possible single-nucleotide polymorphisms (SNPs) present in at least three different sequences were identified and used to build all haplotypes.

Starting from these haplotype sequences, the PCR primers were generated with Hyden [36], a tool for the design of degenerate primers for a given set of DNA sequences. The 454-specific adapters were added to the 5′ end of both forward and reverse primers, together with 10-base molecular barcodes (MID: multiplex identifiers) (Roche 454 Life Sciences, Branford, CT, USA).

### 2.2. Samples

The following samples were analyzed in order to evaluate distinct features of the multiplex PCR-NGS-based system for identification of HR-HPV types and subtypes.

#### 2.2.1. HPV-Positive Cell Lines 

To assess the sensitivity of designed PCR primer set, HPV16 and HPV18 DNA-positive controls were prepared with DNA purified from CaSki and HeLa cells (which contain about 500 and 50 copies of HPV16 and HPV18 genome per cell, respectively) and added at ten-fold dilutions in the DNA carrier obtained from the HPV-negative HEK293 cells, according to the WHO Human Papillomavirus Laboratory Manual [37]. These samples were tested in 20 replicates to set the limit of detection (LoD), defined as the lowest HPV load that allowed PCR amplification and Sanger sequencing with a quality score of ≥20 in 95% of replicate samples.

#### 2.2.2. High-Risk HPV-Positive Genital Swabs

To evaluate the ability of the E6/E7 primer set to identify HR-HPV intra-type variants in clinical samples, we analyzed leftover anogenital swab specimens collected in Specimen Transport Medium (STM, Qiagen, Germantown, MD, USA) at Padova University Hospital from patients with a positive result by the INNO-LiPA HPV Genotyping Extra kit (LiPA, Fujirebio Europe, Gent, Belgium). Specimens included in this study, which were stored at −80 °C until use, were selected from consecutive samples in order to include a representative series of all HR-HPV types. HPV DNA-negative samples were also included as negative controls.

### 2.3. Primer Validation

The primer set reported in this study was designed for broad applications, including Sanger sequencing and NGS protocols. Thus, the performance of the primer set in the identification of HR-HPV intra-type variants was validated by both Sanger sequencing in 36 samples with a single HPV type infection detected by LiPA and by a NGS-based deep sequencing protocol in eight samples with multiple HPV type infection detected by LiPA.

Total DNA was purified from specimens by using the MagNA Pure 96 Viral NA Small Volume Kit on a MagNA Pure 96™ instrument (Roche Diagnostics, Monza, Italy). For PCR amplification, two primer pools composed by forward and reverse primers respectively were prepared, with each primer at 5 µM concentration. A multiplex PCR reaction was performed in a final reaction volume of 50 μL with reaction buffer 10% *v*/*v*, 3 mM MgCl_2_, dNTPs 0.8 mM each, oligonucleotide primers 0.03 μM each and two units of AmpliTaq Gold^®^ DNA Polymerase (ThermoFisher, Waltham, MA, USA). The amplification was carried out with an initial denaturation step of 10 min at 95 °C, followed by 40 cycles of amplification (95 °C, 30 s; 60 °C, 1 min 30 s; 72 °C, 2 min 30 s) and with a final elongation step (72 °C, 7 min) in a MyCycler thermal cycler (Bio-Rad, Hercules, CA, USA).

Sanger sequencing was performed by using the single forward or reverse oligonucleotide primers specific for the HR-HPV type identified by LiPA at a 0.3 μM concentration and a BigDye Terminator v3.1 Cycle Sequencing Kit (ThermoFisher) on an ABI PRISM 3130xl Genetic Analyzer System (ThermoFisher). Electropherograms were visualized through the Chromas Lite 2.1.1 software (Technelysium Pty Ltd., Brisbane, Australia) and aligned to reference sequences from PaVE [38] using Jalview [35].

Deep sequencing was performed on a Roche 454 GS FLX+ instrument (Roche 454 Life Sciences) according to the following Roche GS FLX+ protocols: Amplicon Library Preparation Manual (2014); emPCR Amplification Manual Lib-A-SV (2013); Sequencing Method Manual (2013). Due to the insertion of two distinct barcodes in primer sequences, four pools were generated by mixing equimolar amounts of DNA, each one consisting of two samples. The pools were loaded in separate lanes of the sequencing plate.

### 2.4. Data Processing and Analysis

Sequences obtained by 454 deep sequencing were demultiplexed and primers removed from the 5′ and (if present) 3′ end. The quality control step was performed with USEARCH [34]: sequences that were too short (<250 nt) were removed, while the remaining reads were trimmed starting from the 3′ end until all nucleotide positions with a quality score lower than 10 (corresponding to 10% probability of raw read error) were discarded. Such a cut-off is acceptable because potential errors are counterbalanced by the depth of sequencing. After preprocessing, a total of 471,354 reads were obtained (mean per sample ± standard deviation: 57,073 ± 14,066), with an average length of 713.9 ± 122.4 and an average quality of 34.3 ± 6.8.

For each sample, HPV typing was performed through BLAST [39] alignments of the processed reads against a custom database of HPV genomes downloaded from PaVE [38], similarly to what was done in [22]. To classify a sequence to a certain genotype, at least 90% of read length was required to be aligned with at least 90% of sequence identity, according to HPV typing guidelines [5]. Finally, a consensus sequence was obtained from all reads assigned to the same type within a sample. No indels (insertion and deletions) were detected in consensus sequences.

All the steps not explicitly performed with third-party tools were carried out with custom-made Perl scripts that are available upon request.

The phylogenetic analysis of E6/E7 consensus sequences obtained from the samples, together with the reference sequences for the different genotypes and variants, was performed with the Maximum Likelihood method based on different models for the different species datasets, as reported in figure legends. The analyses were conducted in MEGA6 [40].

Finally, a score based on SNP data was implemented in order to infer viral variants within HPV types. Briefly, SNPs detected in the E6/E7 region of the HR types and associated to each variant were extracted from previous reports [9,41,42,43,44,45,46,47,48]. Then, all the nucleotide positions reported to harbor an SNP were searched in the sequenced samples and, for each SNP, one point was rewarded to all the variants compatible with it. As an example, let a cytosine (C) in position 31 of E6/E7 region be present in variants A and D of HPV type 16, while a thymine (T) is found in variants B and C. For each sample bearing a C in that position, both variants A and D will be scored 1, while no score will be assigned to variants B and C. By repeating this procedure for all SNPs along the sequence, a cumulative score is obtained for each variant and can be used to infer a classification.

High-risk HPV intra-type variant lineages and sublineages were named according to the alphanumeric nomenclature [6].

## 3. Results

### 3.1. Multiplex Polymerase Chain Reaction (PCR) Primers for Characterization of High-Risk Human Papillomavirus (HR_HPV) Intra-Type Variants

By using the bioinformatics pipeline described in the Methods section, a set of primer sequences, including 13 forward and 16 reverse primers, were designed in order to target all HR-HPV types and all their nucleotide variants. Nucleotide sequences and identified targets of the primer set are reported in Table 2. Melting temperature of primers ranged from 59 to 61 °C, mean amplicon size was 817 nt and the maximum delta of amplicon lengths was 88 nt. All the primers were pooled together and used in multiplexing.

The sensitivity of the DNA amplification-sequencing method was assessed by testing replicates of serial dilutions of HPV16 and HPV18 DNA-positive cell lines, *i.e.*, CaSki and HeLa cells, respectively. The LoD for HPV16 and HPV18 was estimated as 900 and 700 genome equivalents in 200 µL STM, respectively. These LoD values were lower than the LoD of LiPA, which was estimated as five genome equivalents per 5 µL of purified nucleic acids for both HPV16 and HPV18, as demonstrated by participation to external quality assurance evaluations promoted by WHO Global HPV DNA typing proficiency studies [49,50].

### 3.2. HPV Typing and Intra-Type Variant Characterization in Single and Multiple Infections

The sequences obtained by Sanger sequencing and the reads obtained by deep sequencing and assigned to each sample were genotyped by means of a BLAST search against a custom database of HPV genome sequences. The multiplex approach of the method, which is designed to target the E6/E7 region of 13 HR-HPV genotypes in the same reaction, allowed detection of the presence of single or multiple infections in all samples.

#### 3.2.1. Sanger Sequencing

A complete agreement was observed between the HPV typing results obtained by LiPA and PCR-Sanger sequencing, as shown in Table 3. However, approximately 10% of cervical swabs positive for high-risk HPVs could not be efficiently amplified by the new PCR primer set, confirming the lower sensitivity of the PCR-sequencing method than LiPA. The lower sensitivity was probably mainly related to the large size of the PCR amplicons generated in our protocol (approximately 800 nt) compared to 65 bp of LiPA amplicons. We did not observe biases in sensitivity associated with specific HPV types, suggesting that the multiplex PCR primer set could amplify the different HR-HPV types with similar efficiency.

Since Sanger sequencing was performed by using only forward and reverse primers specific to the HR-HPV types identified by LiPA as definitely present, HR-HPV types defined as possibly present by LiPA (reported within brackets in Table 3) were not expected by Sanger sequencing. In two cases (samples 890 and 6517), both with HPV58 and possibly HPV52 types as LiPA results, the forward sequencing primer, which was shared between the two HPV types, confirmed only the presence of HPV58. The same happened for sample 007, typed as HPV18 and possibly HPV39 by LiPA and confirmed as HPV18-positive by Sanger sequencing, even though the forward primer could anneal with both HPV18 and HPV39 sequences.

#### 3.2.2. 454 deep sequencing 

A good agreement was also observed between LiPA and the NGS deep-sequencing method (Table 4), with some discrepancies which could be related to the different sensitivity of the two assays for different HPV types. Among the non-HR-HPV types, a high number of reads belonging to HPV34 was found in two samples: this was a non-intended result, due to a good nucleotide pairing with some primers designed for the alpha9 species. This type was not considered in the subsequent analyses.

#### 3.2.3. Characterization of HR-HPV intra-type variants

Two distinct methods were implemented to identify intra-type variants: (1) a phylogenetic analysis and (2) a scoring system based on SNPs.

**Figure 1:**
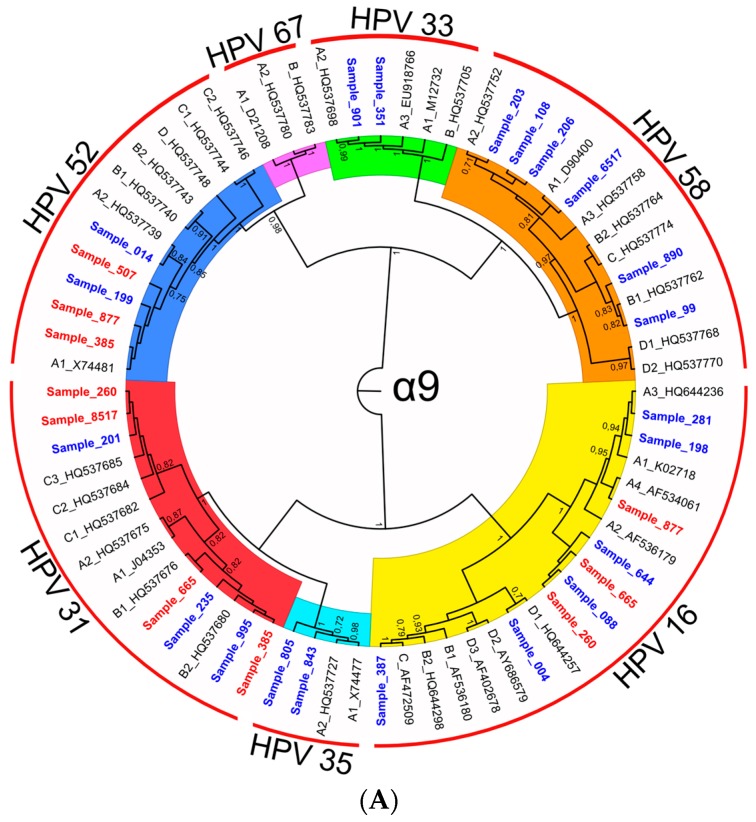

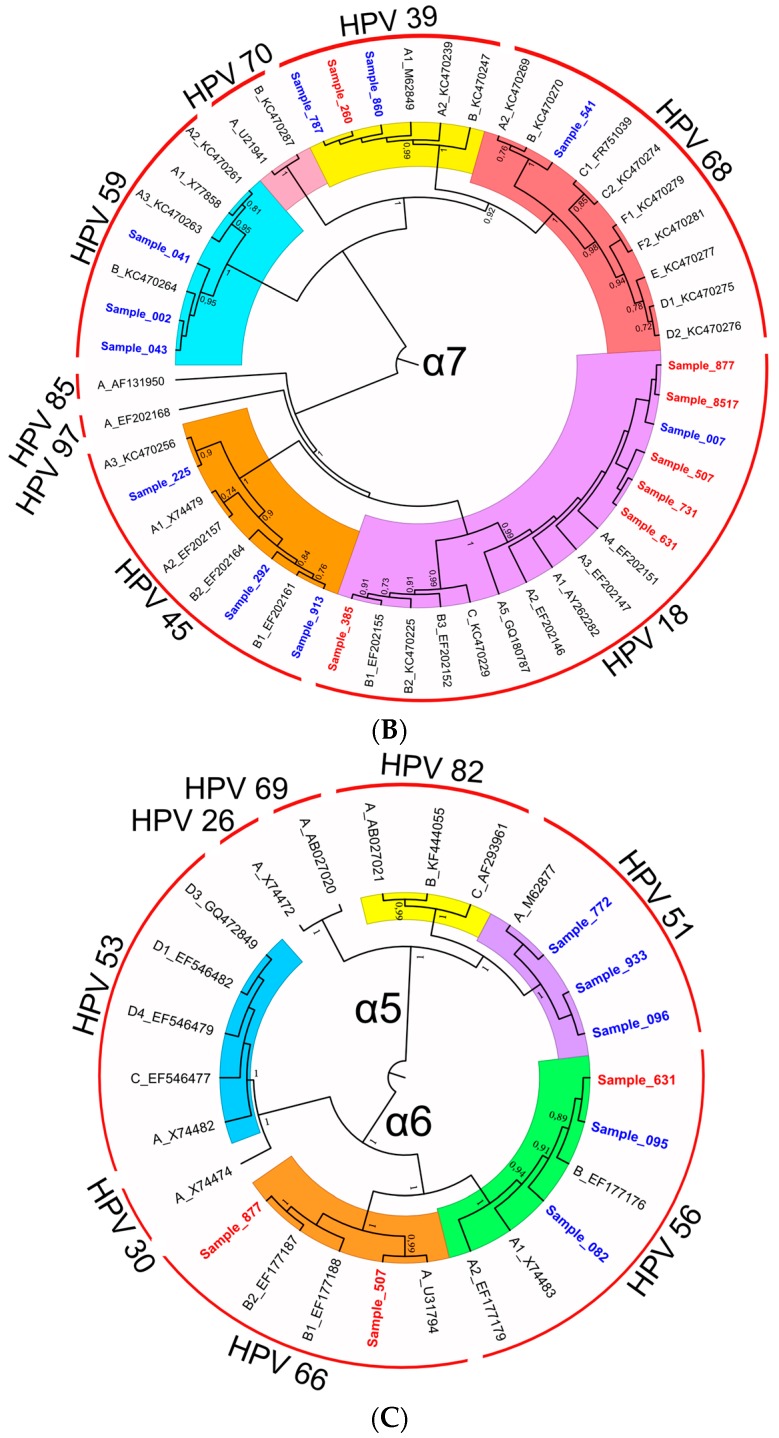
(**A**–**C**) Phylogenetic trees of HPV E6/E7 sequences obtained from clinical samples (red and blue labels for sequences obtained with 454 pyrosequencing and Sanger, respectively) and references (black labels) belonging to the α-9 (**A**); α-7 (**B**); α-5 and alpha-6 (**C**) species inferred by using the Maximum Likelihood method based on the Tamura 3-parameter model [51] (**A**) and the Hasegawa-Kishino-Yano model [52] (**B**,**C**). The percentage of bootstrap replications in which the associated samples clustered together is shown next to the branches (over 1000 iterations). A discrete Gamma distribution was used to model evolutionary rate differences among sites (5 categories: +G, parameter = 0.6006 (**A**), 1.1395 (**B**), 1.6034 (**C**)). Tree branch lengths are proportional to the number of substitutions per site. There were a total of 741, 796, and 695 positions, respectively, in the final dataset of phylogenetic trees (**A**–**C**). Evolutionary analyses were conducted in MEGA6 [40] and displayed with FigTree [53]. Sample labels include sample ID; reference sequence labels include the GenBank accession number and the ID of variant lineages/sublineages (variant lineages are designed as A, B, *etc.*; variant sublineages are designed as A1, A2, *etc.*).

As regards phylogeny, distinct phylogenetic trees were generated for HPV species α-7, α-9, and α-5/α-6 by using the Maximum Likelihood method. The phylogenetic trees included reference sequences for all known variant lineages/sublineages and the HPV sequences obtained from clinical samples (Figure 1). In the figures, HPV types within each species are highlighted with different colors, while numbers close to the nodes represent the percentage of times in which the sequences subsumed by the node were found in the same cluster in bootstrap iterations.

In almost all cases, sample sequences were able to cluster with the reference sequence of a single variant and with a reliable bootstrap value greater than 0.7, as reported in Figure 1 and in Table 5.

**Figure 2:**
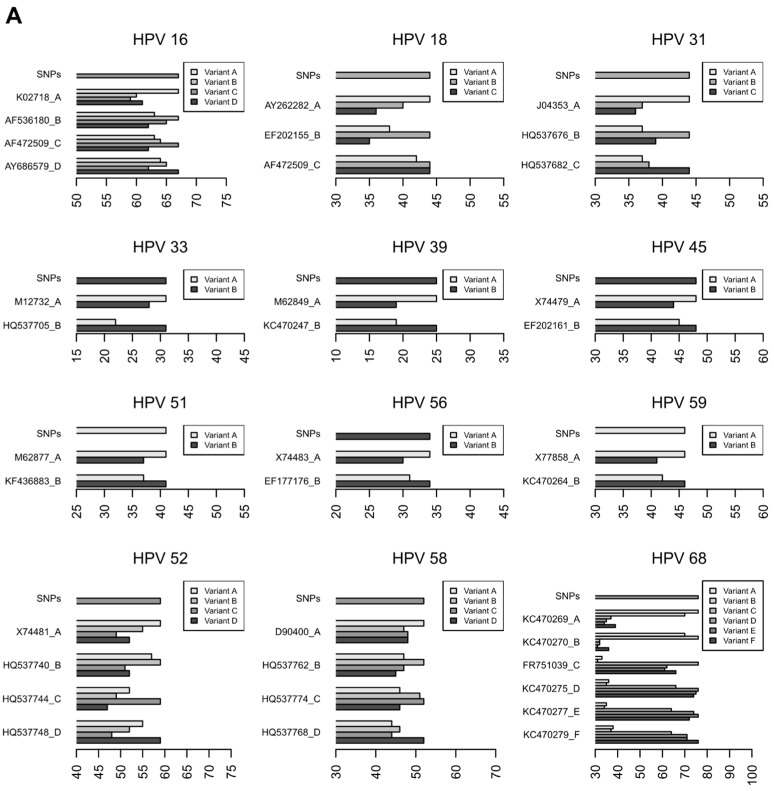

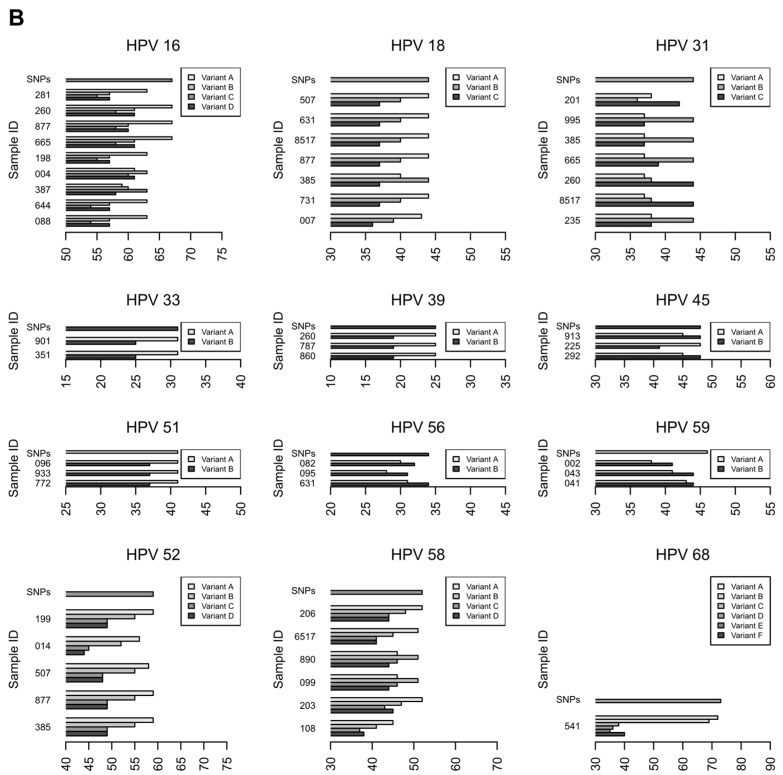
HPV variant assignment based on the SNP score. SNP score is reported in the x axis for the reference variant genomes (**A**) and for the samples sequenced in this study (**B**). HPV sequences detected in samples were assigned to the variant that obtained the highest score. The maximum score achievable for each genotype is reported in each panel and is labeled as “SNPs.”

In order to strengthen and validate the results of HPV subtype classification, we developed an alternative method based on a scoring system of SNPs associated with the different HPV variants. In this method, each nucleotide position in the E6/E7 region presenting variability within an HPV type was evaluated to build a ranking of variants, as detailed in the Methods section. The results are presented in Figure 2A (for variant reference genomes) and Figure 2B (for clinical samples), where the scores obtained by the different variants are reported in the x axis: each sample was assigned to the HPV variant that obtained the highest score.

As shown in Figure 2, some ambiguities occur: e.g., variant C of HPV18 obtained the same score for both lineages B and C (Figure 2A). This happened because the known intra-lineages variability is heterogeneous and there are no specific SNPs of lineage C, while there are SNP positions of lineage B where multiple nucleotides are allowed. From these premises, B *vs.* C and C *vs.* B comparisons are not symmetrical, because B is a superset of C in this particular region of the genome and, inevitably, C reaches the same score when compared to itself and B. Similarly, in some clinical samples, HPV sequences did not obtain the maximum score for any variants, e.g., HPV16 in sample 004. In these cases, possible sources of bias might be considered, such as the incompleteness of available information about lineage variability or the misclassification of the sequences used to build the scoring matrix.

The comparison of the results obtained by the two methods for the classification of HPV variants (*i.e.*, phylogenetic analysis and SNP scoring system) showed an agreement in 52 out of 54 samples (96.3%, Table 5) positive for HPV types for which more than one intra-type variant is known (*i.e.*, only one variant is known for HPV35). The two exceptions included sample 004, which was assigned to HPV16 variant D by phylogeny while the SNP score was unable to discriminate among lineages, and sample 541 which was equally assigned to HPV68 variants A and B by both methods. In the first case, both classifications are quite uncertain, since the bootstrap value for the clade containing sample 004 and HPV16 variant D1 is 0.7 while the SNP score is similar among all HPV16 variants, with no clear separation between the highest score and the others; in the second case, variants A and B of HPV68 are clustered together by phylogeny and obtain a comparable SNP score, suggesting that the E6/E7 region does not provide enough information to discriminate between them.

## 4. Discussion

In this study, we designed and validated a new PCR-NGS-based method for the characterization of HR-HPV intra-type variants in clinical specimens. The set of PCR primers that we developed allows amplification of all HR-HPV types, which are subsequently characterized into viral variants through a double classification based on phylogeny and on a score which considers the SNPs within the E6/E7 region. While many studies have been published on the characterization of the genetic variability of intra-type HPV variants [6,9,10,11,13,14,15,16,17], this is the first attempt to exploit such information to implement a method that combines an NGS-based approach with a bioinformatics pipeline in a new test for HPV variant typing. Proof-of-principle validation experiments were performed on a Roche 454 GS FLX + platform, but this method could be easily adapted to other NGS platforms capable to generate long reads, such as PacBio (Pacific Biosciences, Menlo Park, CA, USA).

Advantages of this method for HPV variant detection as compared to methods reported in the literature are represented by its ability (i) to detect variants of all HR-HPV types in a single reaction; (ii) to detect variants of HR-HPV types in clinical specimens with multiple infections; (iii) being based on sequencing of the full E6/E7 region, to detect amino acid changes in these oncogenes potentially associated with increased transforming activity.

The novel HPV subtyping algorithm based on the SNP score implemented in this study could be used as an alternative to phylogeny for variant classification, since it showed comparable performance. This algorithm can be provided as an online platform, as it only requires *a priori* knowledge of variable sites within the E6/E7 region and their association with HPV intra-type variants. In our evaluation, in some cases, the SNP score method was not able to unambiguously discriminate among HR-HPV intra-type variants. Nonetheless, with the growing availability of sequence data in public databases, an improvement in the discriminating power of the method is expected, helping to overcome the limitations observed for few specific variants. In alternative, an approach based on the detection of haplotypes, instead of independent variable positions, could be adopted.

In the literature, methods for HPV variant detection have been generally designed for testing single HPV types [17,54,55,56,57,58]. By using a multiplexed PCR reaction associated with deep sequencing of amplicons, the NGS-based method reported here allowed to detect and genotype all HR-HPV types directly from clinical specimens without prior knowledge on the HPV types present in the sample. The sensitivity of the method, tested in control HPV16 and HPV18-positive cell lines, was good (up to 900 and 700 copies/reaction respectively), allowing HPV subtyping in clinically relevant infections.

The region targeted in HPV genome for variant detection included the full sequence of the E6 and E7 oncogenes. The most accurate method for the identification of HPV variants/lineages and sublineages is obviously based on full genome sequences [6]. Among the different regions in the HPV genome used for HPV subtyping, such as the NCR, URR, E6/E7, E2, and L1, the URR and E6/E7 regions were shown to provide more accurate subtyping results than other genome regions [44,59], although they generally provide reliable discrimination at lineage but not at sublineage resolution [6,9]. Since the E6 and E7 oncogenes are the key viral factors necessary for tumor formation, nucleotide variants leading to changes in amino acid sequence may be associated with differential risk of transformation and disease progression. Hence, HPV typing and variant detection methods based on the full E6/E7 region offer the advantage of providing genetic information linked to disease pathogenesis. This information might be exploited for the identification of viral genetic traits associated with increased oncogenicity and for the development of antiviral drugs and therapeutic vaccines targeting E6 and E7 oncogenes.

Sanger sequencing, which still represents the gold standard sequencing method, was used in the present study to benchmark the performance of the E6/E7 multiplex primer set in amplifying different HR-HPV types from clinical specimens and identifying their lineages/sublineages. Classification of HR-HPV lineages and sublineages was performed following the nomenclature and the reference HPV genomes proposed by Burk *et al.* [6]. Analysis of a set of clinical samples carrying HR-HPV types as single infection showed that sequencing the target E6/E7 region allowed identification of HPV lineages in most cases. The overall analytical sensitivity of the PCR-sequencing method was lower than LiPA, due to the larger size of the amplicons required for HPV subtyping. However, this method is expected to provide valid results for clinically relevant HR-HPV infections, which are characterized by a relatively high viral load [60]. At variance, analysis of samples characterized by high fragmentation of DNA, e.g., archival formalin-fixed, paraffin-embedded samples, would not be suitable for this method.

After validation of the multiplex PCR protocol by Sanger sequencing, the method was implemented in a 454 deep sequencing protocol. A proof-of-principle sequencing run, performed with eight clinical specimens containing multiple HR-HPV types, showed that the NGS method could correctly identify HPV types and characterize intra-type variants in multiple infections. The inclusion of MID sequences in PCR primers allowed pooling of samples in the sequencing run, thus leading to a reduction of sequencing costs per sample. In our proof-of-principle run, we used a high depth of coverage (*i.e.*, approximately 50,000 reads per sample) that allowed identification of HPV types that were not identified by LiPA probably because of their relatively low concentration in comparison with co-infecting HPV types. A good sensitivity of deep sequencing in the detection of HPV types representing a small proportion (e.g., less than 5% of HPV genome equivalents) in multiple infections was also observed in our previous studies with different primer sets targeting the L1 region [22,23]. Application of a lower depth of coverage would be needed if more samples were pooled, such as in the diagnostic setting, where low costs and high throughput are required. Although obtained from a limited number of samples, our data suggest that a lower depth (e.g., 1000–5000 reads per sample) could be used without loss in sensitivity for clinically relevant HR-HPV infections.

Theoretically, the NGS-based method allows discrimination of different variants of the same HPV type even when they are present in the same sample. This is possible thanks to deep sequencing, since two (or more) different consensus sequences can be obtained from the reads belonging to a certain genotype within a sample. We did not observe such event in the present series of samples, while we detected some cases with multiple infection of variants of the same HPV type in our previous study in a larger series of patients [22].

## 5. Conclusions

In conclusion, this study reports the design and validation of a NGS-based method for characterization of HR-HPV intra-type variants in clinical samples. Notably, the primer set can be adapted to Sanger sequencing and different NGS platforms. This method could be used both for epidemiological studies on the distribution of HPV lineages and for diagnostic purposes. Diagnostic applications may include second line testing in patients with HR-HPV infection, to identify infections associated with increased risk of persistence and disease progression [9,13]. This test could be used together with other biomarkers, e.g., presence of HPV16/18/45, HR-HPV E6/E7 mRNA expression, p16INK4a expression and HPV genome methylation, which have been also associated with an increased risk of progression to malignancy or have been used to define the causative role of HPV in cancer [61,62]. Characterization of HR-HPV variants, especially for HPV types other than HPV16 and HPV18, will be a relevant biomarker in programs for the secondary prevention of HPV-related cancer in the era of prophylactic HPV vaccination.

## Figures and Tables

**Table 1:** High-risk HPV types included in the design of the primer set.

HPV Species	HPV Type	IARC Group	No. of Sequences in Nucleotide Database Covering the HPV E6/E7 Region
α 5	51	1	6
α 6	56	1	126
α 7	18	1	130
	39	1	25
	45	1	104
	59	1	13
	68	2A	92
α 9	16	1	1897
	31	1	276
	33	1	82
	35	1	98
	52	1	363
	58	1	795

**Table 2:** Primers targeting the full E6/E7 region of high-risk HPV types.

HPV Species	Primer Type	Primer Sequence *	HPV Type and Variant ^§^	Amplicon Length (HPV Type)
α 5	F	AACCGAAAAGGGTTATGACCGA	51 (all known variants)	815
	R	TCTGCTGTACAACGCGAAGG		
α 6	F	AGGCAGCTTATTCTGTGTGGA	56 (all known variants)	873
	R	CAGAGTGGGCACGTTACTGT		
α 7	F	AGGGAGTRACCRAAAACGGT	18, 39 (all known variants)	814 (HPV18) 807 (HPV39)
	R	GGAATGCTCGAAGGTCGTCT	18 (all known variants)	
	R	CCCGTGAGGCTTCTACTACC	39 (all known variants)	
	F	TGCAACCAAAAACGGTGCAT	45 (all known variants)	849
	R	TTAGTTGCACACCACGGACA		
	F	GCATGGCACGCTTTGAGG	59 (all known variants)	806
	R	GTTTGCTGCACACAAAGGACA		
	F	GGTCACGACCGAAAACGG	68 (A2, B, D1, D2, E, F1, F2)	815
	R	AGCAGYTSYAGCTTCCGCA	68 (C1, C2, D1, D2, E, F1, F2)	
	F	GKGACCGRAARCGGTCAT	68 (C1, C2)	830
	R	AACAGCTGYTSTAGTGTCCG	68 (A2, B)	
α 9	F	AGGGYGTAACCGAAAACGGT	52, 58 (all known variants)	801 (HPV52) 828 (HPV58)
	R	CCGGGGCACACAACTTGTAA	52 (all known variants)	
	R	ACAGCTAGGGCACACAATGG	58 (A1, A2, A3, B1, B2, D1)	
	R	GCTGTAGGGTTCGTSCTTCA	58 (C, D2)	785
	F	AGGGCGTAACCGAAATCGGT	16 (A1, A2, A3, A4, D1, D2, D3)	830
	R	TGAGAACAGATGGGGCACAC	16 (A1, A2, A3, B1, B2, C, D1, D2, D3)	
	F	TTGMACCGAAACCGGTTAGT	16 (B1, B2, C)	807
	R	RCAGATGGGGCACACAATTC	16 (A4)	
	F	GGTGAACCGAAAACGGTTGG	31 (all known variants)	793
	R	GGGGCACACGATTCCAAATG		
	F	AAGTAGGGTGTAACCGAAAGCG	33 (all known variants)	787
	R	TGCTGTATGGTTCGTAGGTCAC		
	F	ACGGTTGCCATAAAAGCAGAA	35 (all known variants)	827
	R	TCTCTGTGAACAGCCGGGG		

F: forward; R: reverse; * The 454-specific adapters were added to the 5′ end of both F and R primers, together with a 10-base multiplex identifier; ^§^ For each primer, the target genotype is reported; the variants that are covered by the corresponding primer are reported within round brackets.

**Table 3:** Comparison of HPV typing results obtained by Sanger sequencing and LiPA.

Sample ID	LiPA	Sanger Sequencing	
	HPV Type	HPV Type	Scores (Fw, Rw) *
004	HPV16	HPV16	49, 38
088	HPV16	HPV16	48, 42
198	HPV16	HPV16	45, 42
281	HPV16, *HPV53*	HPV16	46, 45
387	HPV16	HPV16	47, 47
644	HPV16	HPV16	47, 46
007	HPV18 (HPV39)	HPV18	36, 15
201	HPV31	HPV31	30, 15
235	HPV31 (HPV52, *HPV54*)	HPV31	30, 36
995	HPV31 (HPV52, *HPV54*)	HPV31	50, 47
351	HPV33, HPV11 (HPV52, *HPV54*)	HPV33	31, 46
901	HPV33 (HPV52, *HPV54*)	HPV33	29, 45
805	HPV35, *HPV44*	HPV35	46, 45
843	HPV35, *HPV54*, *HPV69/71*, HPVX	HPV35	46, 48
787	HPV39, *HPV66*	HPV39	46, 48
860	HPV39	HPV39	49, 49
225	HPV45, *HPV74*	HPV45	45, 31
292	HPV45	HPV45	47, 43
913	HPV45	HPV45	46, 38
096	HPV51	HPV51	37, 43
772	HPV51	HPV51	39, 40
933	HPV51	HPV51	42, 42
014	HPV52	HPV52	37, 41
199	HPV52, HPVX	HPV52	33, 48
082	HPV56	HPV56	15, 30
095	HPV56	HPV56	34, 23
099	HPV58	HPV58	23, 34
108	HPV58	HPV58	16, 26
203	HPV58, *HPV54*	HPV58	43, 35
206	HPV58	HPV58	47, 35
890	HPV58 (HPV52)	HPV58	28, 43
6517	HPV58, *HPV44* (HPV52)	HPV58	26, 14
002	HPV59	HPV59	28, 26
041	HPV59, *HPV54*	HPV59	49, 45
043	HPV59, *HPV43*	HPV59	39, 39
541	HPV68 (HPV39)	HPV68	49, 40

Note: The presence of HPV types reported within brackets is defined as possible according to LiPA. HPVX: Presence of HPV type/s not identifiable by LiPA. HPV types marked in italics are non-high-risk HPV types (not included in the design of the NGS primer set). * Electropherogram quality values calculated by Sequencing Analysis Software 5.3.1 (ThermoFisher): scores were considered high (≥20), medium (≥15 and <20) or low (<15).

**Table 4:** Comparison of HPV typing results obtained by deep sequencing and LiPA.

Sample ID	LiPA	454 Deep Sequencing			
	HPV Type	HPV Type	Total No. Reads (% of the Total)	No. Forward Reads	No. Reverse Reads
8517	HPV18, HPV31, (HPV39, HPV52, *HPV54*)	HPV31	50,660 (93.6)	25,876	24,784
		HPV18	3435 (6.3)	1738	1697
507	HPV18, HPV52, *HPV66*, *HPV69/71*, HPVX, (HPV39)	HPV52	39,860 (72.5)	19,435	20,425
		HPV18	14,913 (27.1)	7629	7284
		*HPV66*	151 (0.3)	81	70
665	HPV16, HPV31, *HPV6*, *HPV69/71,* (HPV52, *HPV54*)	HPV31	69,627 (99.4)	36,230	33,397
		HPV16	408 (0.6)	214	194
731	**HPV16**, HPV18, (HPV39)	HPV18	42,878 (99.9)	23115	19,763
631	HPV18, HPV56, (HPV39, *HPV74*)	HPV18	62,182 (97.4)	33,380	28,802
		HPV56	1564 (2.4)	845	719
385	HPV18, HPV31, (HPV39, HPV52, *HPV54*)	HPV31	45,947 (83.7)	26,291	19,656
		HPV18	8915 (16.2)	5328	3587
		HPV52	22 (0.04)	11	11
877	HPV18, HPV52, *HPV66*, *HPV11*, HPVX, (HPV39)	HPV18	32,879 (68.6)	14,681	18,198
		*HPV34*	13,895 (29)	6048	7847
		HPV52	1012 (2.1)	421	591
		**HPV16**	137 (0.3)	82	55
260	HPV16, HPV39, **HPV52**, *HPV6*, *HPV69/71*, HPVX	HPV39	59,838 (72.9)	29,785	30,053
		*HPV34*	15,042 (18.3)	7274	7768
		HPV16	6145 (7.5)	2921	3224
		**HPV31**	942 (1.1)	423	519

Notes: The presence of HPV types reported within brackets is defined as possible according to LiPA. HPV types marked in italics are non-high-risk HPV types (not included in the design of the NGS primer set). HPV types marked in bold represent discordant results between LiPA and 454 deep sequencing. HPVX: Presence of HPV type/s not identifiable by LiPA.

**Table 5:** HPV type and variant classification through phylogenetic analysis and SNP score.

HPV Type	Bootstrap Value	HPV Intra-Type Variant		Sample IDs
		Phylogenetic Analysis	SNP Score	
16	1.00	A	A	281, 260, 877, 665, 198, 644, 088
16	1.00	C	C	387
16	0.70	D	A, B, C, D *	004
18	0.99	A	A	507, 631, 665, 8517, 877, 731, 007
18	0.91	B	B	385
31	1.00	B	B	995, 385, 665, 235
31	1.00	C	C	201, 631, 260, 8517
33	1.00	A	A	901, 351
35	1.00	A	-**	805, 843
39	0.99	A	A	260, 787, 860
45	0.90	A	A	225
45	0.84	B	B	913, 292
51	1.00	A	A	096, 933, 772
52	0.75	A	A	199, 014, 507, 877, 385
56	0.91	B	B	082, 095, 631
58	0.78	A	A	206, 6517, 203, 108
58	0.96	B	B	890, 099
59	0.95	B	B	002, 043, 041
68	1.00	A, B *	A, B *	541

* Unable to discriminate among indicated lineages. ** The SNP score for HPV35 was not computed since only one variant is known.
